# Detection of Nutritionally Driven Live Weight Changes in Dairy Ewes Using a Walk-over-Weighing System

**DOI:** 10.3390/s26123732

**Published:** 2026-06-11

**Authors:** Mauro Decandia, Marco Acciaro, Giovanni Molle, Andrea Frongia, Maria Sitzia, Maria Gabriella Serra, Andrea Cabiddu, Irene Llach, Eliel González-García, Valeria Giovanetti

**Affiliations:** 1 AGRIS Sardegna, Loc. Bonassai S.S. 291 Sassari-Fertilia Km. 18,6, 07100 Sassari, Italy; macciaro@agrisricerca.it (M.A.); gogiovanni96@gmail.com (G.M.); anfrongia@agrisricerca.it (A.F.); msitzia@agrisricerca.it (M.S.); gserra@agrisricerca.it (M.G.S.); acabiddu@agrisricerca.it (A.C.); vgiovanetti@agrisricerca.it (V.G.); 2 SELMET, INRAE, Montpellier SupAgro, CIRAD, Université Montpellier, 34000 Montpellier, France; irenellach@hotmail.com (I.L.); eliel.gonzalez-garcia@inrae.fr (E.G.-G.)

**Keywords:** walk-over-weighing, live weight monitoring, precision livestock farming, electronic identification, dairy sheep, nutrition, animal welfare

## Abstract

**Highlights:**

- Walk-over-weighing (WoW) detected moderate live weight differences (≈5%) under contrasting nutritional levels.
- WoW captured both short-term (indoor) and sustained (grazing) live weight dynamics.
- Live weight trajectories reflected nutritional constraints and aligned with milk production responses.
- WoW enabled high-frequency, low-labour monitoring for precision livestock farming.
- Data filtering (ORIOLE) ensured robust interpretation of sensor-derived live weight.
- WoW is most effective as a trajectory-based tool when integrated with production and contextual data.

**Abstract:**

Seasonal variability in feed availability in Mediterranean dairy sheep systems can compromise animal performance and welfare, highlighting the need for reliable, high-frequency monitoring tools. Live weight (LW) is a key indicator of nutritional status, but conventional measurements are labour-intensive and poorly suited to dynamic conditions. Walk-over-weighing (WoW) systems integrated with electronic identification (EID) enable automated, continuous, individual-level LW monitoring. This study assessed the sensitivity of a WoW system to detect nutritionally driven LW changes in Sarda dairy ewes under indoor and grazing conditions. Two experiments were conducted: an indoor short-term nutritional challenge involving 24 non-lactating ewes and a grazing trial with contrasting pasture access times involving 48 lactating ewes. In both experiments, the WoW system detected consistent LW differences between nutritional treatments (*p* < 0.001), capturing both short-term responses and sustained LW dynamics. Differences were approximately 5%, indicating that the WoW system was sensitive to nutritionally induced LW variation under the experimental conditions of the present study, before marked changes in body condition score (BCS) became detectable. These results demonstrate that WoW systems can reliably capture LW trajectories in response to nutritional variation. However, LW responses should be interpreted cautiously, as short-term variation may also reflect gut fill and hydration dynamics, and intake information was not fully available at the individual level because some feed intake components were measured at the group level or estimated indirectly. Integrating automated LW data with production and management information may support group-level nutritional decisions and early detection of animals deviating from expected LW trajectories in precision dairy sheep systems.

## 1. Introduction

Mediterranean dairy sheep systems are characterised by marked seasonal variability in forage availability, often exposing grazing ewes to periods of undernutrition [1,2]. Nutritional constraints during late pregnancy, lactation, or mating may impair milk production, metabolic homeostasis, reproductive performance, and flock efficiency [3,4,5,6]. Temporary nutritional constraints may also arise from management-related factors such as regrouping, inaccurate feed allocation, or inadequate diet quality [7,8]. These conditions highlight the need for reliable tools to monitor nutritional status under variable production conditions.

Live weight (LW) is a practical and integrative indicator of nutritional status and body reserve dynamics and is therefore relevant for both production performance and welfare assessment in dairy ewes [9,10]. Accordingly, monitoring LW dynamics provides a direct link between nutritional management, animal performance, and welfare outcomes. During the transition from late pregnancy to early lactation, ewes commonly mobilise body reserves to sustain milk production, and excessive LW loss may indicate an imbalance between nutrient intake and production demand [11]. Prolonged LW decline is associated with reduced performance and may reflect nutritional insufficiency and/or health challenges. Moreover, LW and body condition at the end of lactation reflect body reserve status and are useful indicators for nutritional management and long-term animal performance [5,12,13]. Optimising LW trajectories has also been linked to improved whole-farm profitability [12,13].

Despite its importance, LW is rarely measured at high frequency in commercial dairy sheep systems because conventional weighing is labour-intensive, time-consuming, and potentially stressful for animals [14]. Conventional static weighing is also episodic and therefore poorly suited to detecting rapid changes in nutritional status or describing individual LW trajectories over time. In grazing systems, short-term LW variability related to gut fill and hydration further complicates interpretation, making repeated measurements or data averaging necessary to improve measurement precision [14]. Precision livestock farming technologies offer new opportunities to overcome these limitations by enabling continuous and automated animal monitoring [15,16]. In particular, walk-over-weighing (WoW) systems integrated with electronic identification (EID) enable automated, high-frequency, individual-level LW monitoring with minimal animal handling, allowing the analysis of LW dynamics rather than single-point measurements [17,18,19].

WoW platforms have been tested in sheep under different production systems, generally showing good agreement with static weighing systems while highlighting the importance of data filtering procedures to manage noise and outliers [20,21,22,23]. Under grazing and commercial conditions, voluntary animal traffic, unstable or simultaneous crossings, and behavioural variability may generate tracking errors and biologically implausible records, highlighting the importance of robust filtering and data-processing procedures. Recent validation studies have demonstrated the value of Kalman-filter-based approaches, such as the ORIOLE/kfino framework, for improving the reliability and interpretation of WoW-derived LW trajectories [24].

However, experimental evidence on the ability of WoW systems to detect moderate, biologically relevant LW changes associated with nutritional variation remains limited, particularly under contrasting management conditions such as indoor feeding and grazing. This limits the interpretation of WoW data for practical decision-making in commercial systems.

Therefore, the aim of this study was to evaluate the sensitivity of a WoW system to detect moderate LW changes induced by different nutritional levels in Sarda dairy ewes managed either indoors (dry ewes) or at pasture (lactating ewes). By addressing this gap, the study contributes to the assessment of automated LW monitoring as a sensor-based tool to support nutritional management and welfare-oriented decision-making in dairy sheep systems.

## 2. Materials and Methods

### 2.1. Study Site and Ethical Statement

The study was conducted at the Bonassai experimental farm of the Agricultural Research Agency of Sardinia (AGRIS Sardegna; Italy; 40°40′16.215″ N, 8°22′0.392″ E; 32 m a.s.l.). Two experiments were conducted under contrasting production systems: Experiment 1 (E1) under indoor (stabled) conditions and Experiment 2 (E2) under grazing conditions. All procedures were conducted in accordance with European Union Directive 2010/63/EU on the protection of animals used for scientific purposes [25]. The study was approved by the Ethical Committee for Animal Experimentation (O.P.B.S.A.) of the University of Sassari and AGRIS Sardegna (approval no. 52682, 6 May 2021).

### 2.2. Experiment 1 (E1): Stabled Ewes

#### 2.2.1. Animals, Housing, and Experimental Design

Experiment 1 was conducted in spring 2022 (late April to early June) using 24 non-pregnant and non-lactating Sarda ewes, including 12 young (2–3 years) and 12 mature ewes (4–6 years). Animals were allocated to two groups balanced for age (2.7 ± 0.3 years, mean ± SE), live weight (LW; 55.5 ± 0.9 kg) and body condition score (BCS; 2.95 ± 0.03).

Each group was housed in a separate bedded pen, with free access to water and access to an adjacent outdoor paddock. The experimental period consisted of four consecutive phases:

(i) Pre-adaptation (2 weeks), including regrouping and habituation to the feeding system and weighing circuit;
(ii) Adaptation (2 weeks), during which a common diet was provided;
(iii) Nutritional challenge (2 weeks), during which contrasting feeding levels were applied;
(iv) Recovery (1 week), during which animals returned to the baseline diet.

#### 2.2.2. Feeding Management

During the pre-adaptation and adaptation phases, both groups received a ration formulated to supply approximately 110% of maintenance energy requirements [3] for Sarda ewes of similar body weight and physiological status. The diet consisted of pelleted concentrate (0.1 kg/ewe/day), offered individually in the milking parlour, together with a mixed feed (0.9 kg/ewe/day) and lucerne hay (0.5 kg/ewe/day), both provided at the group level in the pen. The use of the milking parlour, despite the animals being non-lactating, allowed accurate measurement of individual concentrate through individual feeding stations, which would not have been feasible under group-feeding conditions. The mixed feed was a fibre-based ration including sugar beet pulp, cereal grains and chopped forage, supplemented with protein meals and minerals.

During the nutritional challenge phase, feeding levels (FL) were adjusted to approximately 70% (Low, L) and 140% (High, H) of maintenance requirements by modifying the quantities of mixed feed and hay, while the amount of concentrate remained constant. Feeding schedules, including timing and feed delivery, were kept consistent across all experimental phases. Individual concentrate intake was recorded in the milking parlour, whereas intake of mixed feed and hay was measured at the group level by weighing feed offered and refusals.

#### 2.2.3. Walk-over-Weighing (WoW) System and Data Acquisition

**Figure 1:**
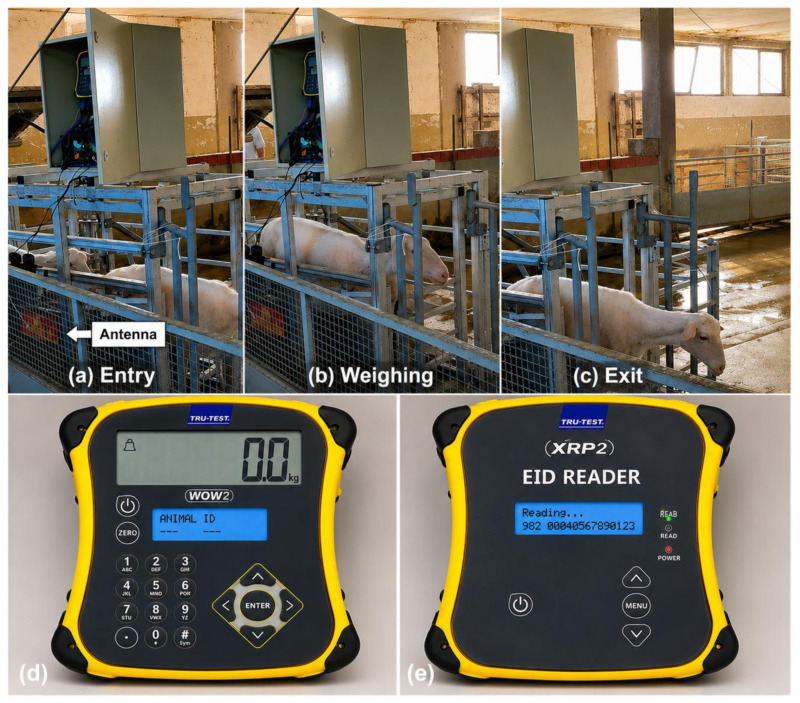
Walk-over-weighing (WoW) system for automated live-weight monitoring of dairy ewes. The sequence shows (**a**) animal identification via the EID antenna at entry, (**b**) live-weight acquisition on the weighing platform, and (**c**) exit from the system. Panels (**d**) and (**e**) show the main hardware components used for data acquisition, namely the WoW2 weigh scale indicator and the XRP2 EID reader, respectively. During the experiments, animal passage through the platform was guided to ensure reliable identification and live-weight acquisition.

Live weight was recorded using a walk-over-weighing (WoW) platform (Maréchalle Pesage, Chauny, France) integrated with electronic identification (EID) [20]. Each ewe was equipped with a ruminal EID bolus (Datamars HDX, Datamars SA, Bedano, Switzerland), detected by an antenna connected to a TRU-TEST XRP2 reader and WoW2 indicator (Tru-Test Ltd., Auckland, New Zealand) (Figure 1).

The objective of the present study was not to perform a formal technical validation of the weighing accuracy of the WoW system, but rather to evaluate its ability to detect biologically meaningful live-weight changes associated with contrasting nutritional conditions. Accordingly, the study focused on the sensitivity of WoW-derived live-weight trajectories to nutritional challenges rather than on technical performance metrics such as accuracy, repeatability, systematic bias, or agreement with reference scales.

To ensure consistent data acquisition under controlled experimental conditions, animals were guided through a fixed circuit including the weighing platform, positioned in the stall, during routine management operations (feeding and handling). During a two-week pre-adaptation phase, animals were progressively habituated to the weighing circuit and guided passage through the WoW platform to minimise stress, hesitation, and behavioural disturbances during weighing. During this period, animals were regularly encouraged to cross the platform under controlled conditions to facilitate voluntary and stable passages.

During the experimental period, ewes were guided to cross the WoW platform three times per day in the morning as part of the daily management routine. Animal flow was controlled to allow one ewe at a time to pass through the platform, thereby reducing crowding effects, simultaneous crossings, and unstable measurements associated with abrupt movements. This controlled passage was implemented to ensure sufficient data density and minimise missing, duplicated, or biologically implausible records, rather than to simulate commercial farm conditions. Standardised passage conditions improved repeatability and comparability of measurements across animals.

#### 2.2.4. Additional Measurements

Feed samples were analysed for dry matter (DM), crude protein (CP), ether extract (EE), neutral detergent fibre (NDF), acid detergent fibre (ADF) and acid detergent lignin (ADL) [26], and in vitro dry matter digestibility (IVDMD) using the pepsin–cellulase method [27]. The chemical composition and IVDMD of feedstuffs were determined at three time points during the experiment (beginning, mid and end of the trial) to account for temporal variability in feed quality. Dietary energy (UFL/kg DM) and metabolizable protein (MP/kg DM) were calculated [3].

Body condition score was assessed fortnightly using a 1–5 scale [28]. Health events and behavioural anomalies potentially affecting weighing were recorded.

### 2.3. Experiment 2 (E2): Grazing Ewes

#### 2.3.1. Animals and Experimental Design

Experiment 2 was conducted in spring 2023, from mid-April to mid-June (nine weeks in total). Two groups of adult lactating ewes (24 animals each) were selected based on homogeneous characteristics, including age (3.5 ± 0.8 years; mean ± SE), lambing date (19 February 2023 ± 7 days), stage of lactation (51 ± 7 days in milk), milk yield (2.31 ± 0.07 kg/ewe/day), LW (49.9 ± 0.9 kg) and BCS (2.7 ± 0.03). Ewes were machine-milked twice daily throughout the study. None of the ewes had previous experience with the WoW system.

#### 2.3.2. Feeding and Grazing Management

Ewes had part-time access to pasture and received supplementary feeding: concentrate (0.400 kg/head/day) in the milking parlour and hay (0.720 kg/head/day) in the pen.

Two grazing treatments were applied based on different pasture access times:
AT6 (6 h/day pasture access; designed to meet nutritional requirements);
AT2 (2 h/day pasture access; restricted intake conditions).

Treatments were applied at the group level without true group replication. Therefore, potential group effects cannot be fully separated from treatment effects, and results should be interpreted with appropriate caution. The grazing area (approximately 1.8 ha), consisting of a mixture of Italian ryegrass and Berseem clover, was subdivided into 12 plots using temporary electric fencing and managed under rotational grazing (7 days per plot). Water was available both at pasture and in the housing area.

Herbage mass was measured weekly using quadrat sampling, and botanical composition was determined by manual separation.

Herbage intake was estimated using an established predictive equation [29], recognising that this represents an indirect estimate rather than a direct measurement of individual intake. Individual concentrate intake was recorded in the milking parlour, whereas intake of hay was measured at the group level by weighing feed offered and refusals.

#### 2.3.3. Walk-over-Weighing (WoW) System and Data Acquisition

The same WoW system used in E1 was employed (Figure 1). During the adaptation phase, animals were progressively habituated to cross the WoW platform, positioned in the stall close to the milking parlour, in order to facilitate voluntary passage and reduce behavioural disturbances during weighing. During the experimental period, ewes crossed the platform twice daily after morning milking and before pasture access. Animal flow was controlled to ensure reliable data acquisition and measurement consistency; these conditions do not fully reflect the variability in animal traffic and data density typically encountered under commercial grazing systems with voluntary passage through WoW platforms.

#### 2.3.4. Additional Measurements

Feed chemical composition (DM, CP, EE, NDF, ADF, ADL) and in vitro dry matter digestibility (IVDMD) were determined using standard analytical procedures [26,27]. For hay and grazed herbage (hand-plucked samples), composition and IVDMD were estimated using a NIRS system (NIRS 5000, FOSS NIRSystems, Silver Spring, MD, USA) calibrated against reference methods. Analyses were performed at three time points during the experiment (beginning, mid and end of the trial) to account for temporal variability in feed quality. Dietary energy (UFL/kg DM) and metabolizable protein (MP/kg DM) were calculated [3].

Individual milk yield was recorded fortnightly. Milk samples were analysed for fat and true protein (N × 6.38) using an infrared analyser (Milkoscan, FOSS, Hillerød, Denmark). Fat and protein corrected milk yield (FPCM) was calculated according to [30]. BCS was assessed fortnightly using a 1–5 scale [28].

### 2.4. Data Processing and Statistical Analysis

Raw WoW data were screened to remove records without valid EID identification. Data were subsequently filtered using the ORIOLE web application [24], which integrates the kfino algorithm based on a Kalman-filtering approach adapted for WoW data processing [31]. The algorithm uses the temporal trajectory of individual LW records to identify and remove impulsive outliers and biologically implausible measurements typically associated with abrupt movements, unstable passages, or simultaneous crossing on the platform. After filtering, approximately 88–89% of records were retained across experiments. Valid LW records were then aggregated to calculate daily mean LW values for each ewe. This approach has been previously validated under different sheep farming conditions for improving the reliability of automated WoW measurements [24]. All valid WoW passages retained after filtering were used for analysis. When multiple valid records were available for the same ewe on a given day, they were aggregated to calculate a daily mean LW value, which was subsequently used for statistical analyses. The number of retained valid records per ewe depended on the experiment and passage frequency imposed by the experimental design. Individual ewes were considered the experimental unit for live weight, body condition score and production traits. However, intake of mixed feed and hay was measured at the group level and therefore not available at the individual level. Consequently, intake-related results should be interpreted with caution, as individual variability and potential social effects could not be accounted for. LW, BCS, and milk traits were analysed using linear mixed models, including feeding treatment, time (week), and their interaction as fixed effects. Ewe was included as a random intercept effect to account for repeated observations collected on the same animal over time. Repeated measurements were modelled using an autoregressive covariance structure [AR(1)], and degrees of freedom were adjusted using the Kenward–Roger method. Initial values were included as covariates when appropriate. Additional models were used to explore associations between LW and intake-related variables. These analyses were intended to evaluate the biological sensitivity of WoW-derived live weight to nutritional variation and should not be interpreted as a formal validation of weighing accuracy or measurement precision. In E2, principal component analysis (PCA) was applied to address multicollinearity between milk yield and intake-related variables when evaluating the relationship between live weight and nutrient intake. PCA was performed on standardised milk yield and DMI variables. Milk yield and DMI were moderately correlated (r = 0.518). The first principal component (PC1) explained 75.9% of the total variance and represented the variation shared between milk production and feed intake. The second principal component (PC2) explained 24.1% of the variance and was orthogonal to PC1. The loading coefficients for PC1 were 0.707 for milk yield and 0.707 for DMI, whereas PC2 contrasted the two variables with loadings of −0.707 for milk yield and 0.707 for DMI. To facilitate biological interpretation, the sign of PC2 was reversed, and the resulting variable was defined as Milk_PC2, such that higher values represented greater milk production relative to feed intake. Consequently, Milk_PC2 can be interpreted as an intake-independent descriptor of milk production efficiency, identifying ewes producing relatively more milk than expected for a given level of feed intake. Milk_PC2 was therefore included as a predictor in the linear mixed models.

Statistical analyses were performed using SAS software (version 9.4; SAS Institute Inc., Cary, NC, USA) [32].

## 3. Results

### 3.1. Experiment 1 (E1): Stabled Ewes

#### 3.1.1. Feed Intake and Nutritional Treatments

The chemical composition and in vitro dry matter digestibility (IVDMD) of the feedstuffs are reported in Table 1 (means ± SE), while weekly averages of dry matter intake (DMI), energy intake (UFLI), and protein intake (CPI and MPI) are presented in Table 2. During the nutritional challenge phase (W3–W4), estimated intake levels differed markedly between treatments, reflecting the imposed feeding levels. Ewes in the high feeding level (H) group showed significantly higher DMI, UFLI, CPI and MPI compared to the low feeding level (L) group (feeding level effect: *p* < 0.001 for all traits). Conversely, during the adaptation (W2) and recovery (W5) phases, intake values were slightly higher in L than in H (Table 2). Experimental week significantly affected all intake variables (*p* < 0.001), with the main differences occurring between the adaptation/recovery and challenge phases (Table 2). These patterns reflect the imposed feeding levels and confirm the effectiveness of the nutritional treatments.

#### 3.1.2. Live Weight and Body Condition

**Figure 2:**
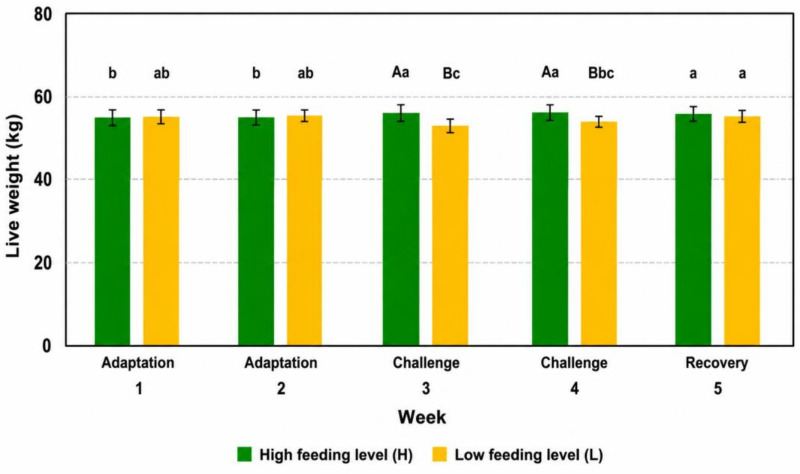
Weekly live weight (LW, kg) of ewes subjected to high (H) and low (L) feeding levels during the indoor experiment. The study comprised an adaptation phase (weeks 1–2), a nutritional challenge phase (weeks 3–4), and a recovery phase (week 5). Values are least-square means ± SEM. Different uppercase letters indicate significant differences between feeding treatments within the same week, whereas different lowercase letters indicate significant differences among weeks within the same treatment (*p* < 0.05).

Live weight (LW) results obtained from the WoW system are presented in Figure 2. When averaged over the entire experimental period, feeding level did not significantly affect LW (*p* = 0.07). However, both experimental week (*p* < 0.001) and the interaction between feeding level and week (*p* < 0.001) were significant. Differences between treatments were evident during the nutritional challenge phase, with H ewes showing higher LW than L ewes in both W3 and W4. Within-group patterns indicated that LW increased in H ewes from adaptation to challenge and remained elevated during recovery, whereas L ewes exhibited a marked LW decrease in W3, followed by partial recovery in W4 and W5.

The temporal pattern of LW responses throughout the adaptation, challenge, and recovery phases is additionally illustrated in Supplementary Figure S1. These patterns indicate that LW responded consistently to the imposed nutritional treatments. Body condition score (BCS) was not affected by feeding level but increased significantly over time (week effect: *p* < 0.001) in both groups.

### 3.2. Experiment 2 (E2): Grazing Ewes

#### 3.2.1. Pasture Characteristics and Feed Composition

Herbage biomass availability was high and did not differ between treatments (*p* > 0.05; Table 3). Based on an assumed utilisation rate of 50%, daily herbage availability per ewe was estimated to be comparable between groups. However, when expressed per hour of access, herbage availability was higher in the AT2 group. Pasture composition was dominated by Italian ryegrass in both treatments, with a higher proportion observed in AT2 (Table 3). Conversely, the proportion of other species was greater in AT6. Feed chemical composition is reported in Table 4. Grazed herbage in AT2 showed numerically higher crude protein (CP), lower neutral detergent fibre (NDF), and higher acid detergent lignin (ADL), although differences were not statistically significant.

#### 3.2.2. Intake and Nutritional Level

Estimated herbage dry matter intake was higher in ewes with 6 h pasture access (AT6) compared to those with 2 h access (AT2) across all experimental weeks. Accordingly, total intakes of DMI, UFLI, CPI, and MPI were also higher in AT6 than AT2 (*p* < 0.001; Table 5). Experimental week also affected all intake traits (*p* < 0.001), with a progressive decline over time in both groups. The significant interaction between access time and week (*p* < 0.001) indicates differences in the magnitude and pattern of this decline between treatments.

#### 3.2.3. Live Weight and Body Condition

**Figure 3:**
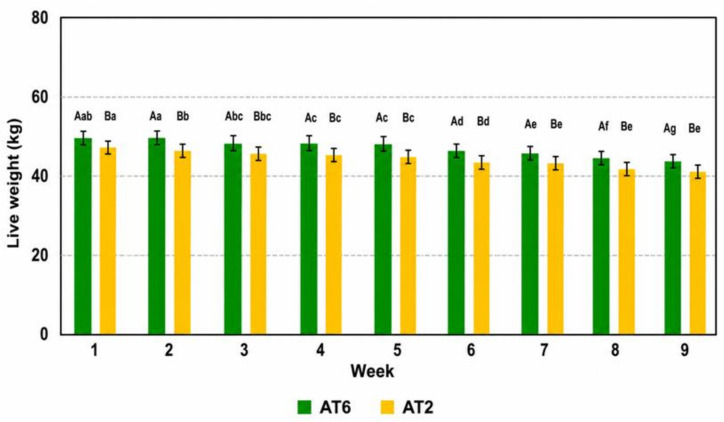
Weekly live weight (LW, kg) of lactating ewes monitored using the walk-over-weighing (WoW) system under two pasture access treatments (AT6: 6 h/day; AT2: 2 h/day) during the grazing experiment. Values are least-square means ± SEM and represent daily means derived from filtered WoW records. Different uppercase letters indicate significant differences between pasture access treatments within the same week, whereas different lowercase letters indicate significant differences among weeks within the same treatment (*p* < 0.05).

Across the 9-week experimental period, AT6 ewes maintained higher LW than AT2 ewes (treatment effect: *p* < 0.001; Figure 3). The average LW difference between treatments was approximately 2.1 kg (≈5%). LW decreased over time in both groups (week effect: *p* < 0.001), with a significant interaction between access time and week (*p* < 0.001). The temporal pattern of LW responses throughout the grazing period is additionally illustrated in Supplementary Figure S2. BCS was not affected by pasture access time but declined significantly over time (week effect: *p* < 0.001).

#### 3.2.4. Milk Production

Milk yield and fat- and protein-corrected milk (FPCM) were affected by experimental week (*p* < 0.001) and pasture access time (milk: *p* < 0.01; FPCM: *p* < 0.05), with significant interactions (milk: *p* < 0.01; FPCM: *p* < 0.001, Table 6). Differences between treatments were most evident during the mid-experimental period, when AT6 ewes produced more milk and FPCM than AT2, whereas values converged towards the end of the experimental period. Milk fat and protein concentrations were affected by week (*p* < 0.001) but not by pasture access time (fat: *p* = 0.11; protein: *p* = 0.88), and no significant interaction was observed (Table 6).

### 3.3. Relationship Between Intake, Live Weight, and Milk Yield

Across both experiments, live weight (LW) was positively associated with intake-related variables (Table 7). All estimated slopes were highly significant (*p* < 0.001), indicating that higher LW values were generally associated with higher estimated nutrient intake. Specifically, DMI increased by 0.023 kg/day per kg of LW, UFLI by 0.024 UFL/day per kg of LW, and CPI and MPI by 4.52 and 4.07 g/day per kg of LW, respectively. These relationships should be interpreted with caution because intake data were not fully available at the individual level: concentrate intake was individually recorded, whereas forage intake was measured at the group level and herbage intake was individually estimated in Experiment 2. Model fit was satisfactory, with marginal R^2^ ranging from 0.73 to 0.81 and conditional R^2^ from 0.87 to 0.94, indicating that a large proportion of the variation was explained by fixed effects, with additional variance accounted for by the random animal effect.

Within Experiment 2, the selected models included live weight (LW), week of lactation, and Milk_PC2, the second principal component derived from PCA of milk yield and DMI and representing milk production variation independent of intake-related variables (Table 8). The association between LW and intake-related variables differed among traits. LW was significantly associated with CPI and MPI (*p* < 0.05), whereas no significant association was observed for DMI or UFLI (*p* > 0.05). Week of lactation and Milk_PC2 were significant predictors across all intake traits (*p* < 0.001). Model fit was satisfactory, with marginal R^2^ ranging from 0.59 to 0.76 and conditional R^2^ from 0.72 to 0.94.

## 4. Discussion

This study was designed to evaluate the sensitivity of a walk-over-weighing (WoW) system to detect moderate live weight (LW) changes associated with contrasting nutritional levels in dairy ewes under indoor and grazing conditions. The primary objective was to assess the ability of the WoW system to detect biologically relevant LW variation under contrasting nutritional conditions rather than to perform a technical validation of its absolute weighing accuracy under commercial conditions. Although periodic static LW measurements were used as operational reference values during the experiments, formal agreement analysis between WoW and static scales (e.g., Bland–Altman analysis or repeatability metrics) was beyond the scope of the present study. Across both experiments, ewes exposed to lower nutritional levels consistently exhibited lower LW, and the WoW system was able to capture these differences under contrasting management contexts. The agreement between imposed or estimated nutritional contrasts and LW trajectories supports the ability of the system to detect biologically meaningful LW variation rather than random measurement noise. Therefore, the present findings should be interpreted primarily as evidence of the biological sensitivity of the WoW system rather than as a comprehensive validation of its absolute weighing accuracy under commercial farming conditions. However, feed intake was not directly measured at the individual level in all experimental conditions, and part of the nutritional exposure was assessed through group-level measurements or predictive estimates. Consequently, the association between LW changes and the nutritional status of individual animals should be interpreted with caution.

### 4.1. Sensitivity of WoW to Nutritional Variation

In Experiment 1, LW differences between feeding levels were detectable but relatively moderate. This is consistent with the lower nutrient requirements of non-lactating ewes, the short duration of the nutritional challenge, and the reduced energetic demands of indoor housing. Under these conditions, changes in nutrient intake are expected to affect gastrointestinal fill and body reserves, with LW responding more rapidly than body condition score (BCS), which typically reflects longer-term changes. The divergence of LW during the challenge phase and convergence during recovery supports the reliability of the system.

LW increases observed in the high-feeding group may reflect increased rumen fill combined with limited body reserve changes, whereas the restricted group showed a rapid LW decline followed by incomplete recovery, consistent with the asymmetry between mobilisation and repletion of body reserves. Metabolic adaptations to undernutrition may contribute to this pattern [33], although this interpretation remains speculative because metabolic indicators were not measured. Additionally, inclusion of younger animals potentially still undergoing growth may have increased variability in LW responses. These interpretations remain hypothetical, as body composition, rumen fill and metabolic indicators were not directly measured in this study.

Interpretation of intake patterns in E1 should consider that forage and mixed feed intake was measured at the group level. Group-based measurements may mask individual variability arising from competition and feeding behaviour, particularly under restricted conditions. Social dynamics may therefore contribute to heterogeneous nutritional status within groups [34,35], potentially explaining discrepancies between intake estimates and individual LW trajectories. In addition, the experimental design does not allow full separation of treatment and group effects, as treatments were applied at the group level without replication; therefore, results should be interpreted as indicative rather than definitive.

### 4.2. WoW Performance Under Grazing Conditions

In Experiment 2, restricting pasture access time resulted in sustained differences in intake and LW. Despite expected behavioural compensation (i.e., increased intake rate under limited access), ewes with restricted access (AT2) consistently showed lower intake and LW than those with extended access (AT6). This indicates that access time remained the dominant constraint on intake, even under adaptive grazing behaviour [29].

The persistence of LW differences over the nine-week period indicates the ability of the WoW system to capture longer-term nutritional effects under grazing conditions. The average LW difference between treatments was approximately 2.1 kg (≈5%). Concurrent differences in milk yield and fat- and protein-corrected milk (FPCM), particularly during mid-lactation, further support the biological relevance of LW trajectories. The convergence of milk production towards the end of the experiment likely reflects the combined effects of advancing lactation stage and declining intake. The observed decline in LW and BCS in both treatments highlights the influence of physiological stage and seasonal factors beyond experimental treatments. The strong contribution of Milk_PC2 to intake models, representing milk yield variation independent of intake-related effects, suggests that milk production demand is a major driver of variability. This supports a multifactorial regulation of intake involving LW, production level, and stage of lactation.

### 4.3. Implications for Precision Livestock Farming

These findings support the use of LW as a practical and scalable indicator of nutritional status with direct implications for welfare-oriented management. Nutritional imbalances can affect animal health through metabolic and immune pathways [36,37], and are particularly relevant in Mediterranean systems characterised by seasonal variability in feed availability [38,39]. The WoW system enables high-frequency, low-labour LW monitoring, allowing detection of deviations from expected LW trajectories. Its value lies primarily in trajectory-based interpretation over time rather than in single measurements. This is especially relevant in systems where group feeding or grazing management may result in heterogeneous individual responses. The guided multiple daily crossings implemented in this study were designed to ensure data completeness under controlled experimental conditions and may not fully reflect commercial farm settings, where voluntary animal movement would be expected, particularly in housed systems.

### 4.4. System Performance and Data Processing Considerations

The WoW system detected consistent differences in LW of moderate magnitude (≈5%) between nutritional treatments in both experiments. However, direct comparison between experiments is limited due to differences in experimental design, duration, and physiological stage. Reliable interpretation of WoW data depends on data quality and appropriate filtering. Approximately 10–12% of raw records were removed during processing, which is relatively low compared to less controlled or commercial systems. This likely reflects the structured animal flow and guided crossing design adopted in this study. Data filtering tools such as ORIOLE are therefore essential to ensure that LW estimates reflect biological variation rather than artefacts [24,31].

### 4.5. Potential for Intake Estimation and Modelling

Beyond LW monitoring, WoW systems show potential to support inference on feed intake and efficiency. Short-term LW variation has previously been used to estimate individual intake in grazing sheep [20]. Under restricted grazing, repeated measurements before and after grazing may provide a simplified proxy for intake estimation, although confounding factors such as hydration and excretion must be considered. Cross-experiment modelling showed that LW was associated with intake-related variables. However, these relationships should be interpreted with caution, as intake was only partially available at the individual level and partly derived from group-level measurements or predictive estimates. In lactating ewes, inclusion of Milk_PC2, representing milk yield variation independent of intake, improved model performance and allowed separation of intake-driven and production-driven effects. This highlights the importance of accounting for production demand when modelling intake-related responses. These findings are consistent with precision nutrition approaches, where LW, production data, and physiological stage are integrated to improve feeding strategies.

### 4.6. Limitations and Practical Implications

Despite its potential, WoW has limitations. The results do not support the definition of universal LW thresholds for diagnosing undernutrition, as LW responses depend on physiological stage, diet type, gut fill dynamics, and temporal scale. Furthermore, WoW alone cannot distinguish between changes in body reserves and short-term variation in gut fill or hydration without complementary information. Therefore, short-term LW fluctuations should not be interpreted exclusively as indicators of body reserve mobilisation or accretion, particularly when complementary measurements such as body condition score, metabolic indicators, or body composition assessments are not available.

Moreover, WoW cannot replace direct intake measurements, particularly under grazing conditions where intake estimation remains challenging. Its primary value lies in the early detection of deviations from expected LW trajectories, particularly when integrated with other data streams such as milk production.

An additional limitation of the present study is that treatments were applied at the group level without replicated groups. Consequently, part of the observed variation may reflect group-specific effects that could not be fully disentangled from the nutritional treatment itself. Therefore, the present results should be interpreted as evidence of WoW sensitivity under the tested experimental conditions rather than as definitive quantification of treatment effects.

The present results were obtained under controlled experimental conditions with guided animal passage through the WoW platform to ensure sufficient data density and measurement stability. Under commercial conditions, where animal passage would be more voluntary, lower passage frequency and greater variability in data acquisition could occur, potentially affecting monitoring performance. Therefore, further validation under fully commercial farming conditions remains necessary. Consequently, extrapolation of the present results to commercial systems should be made with caution.

## 5. Conclusions

The walk-over-weighing (WoW) system demonstrated the ability to detect moderate live weight (LW) changes associated with contrasting nutritional levels in Sarda dairy ewes under both indoor and grazing conditions. Under controlled indoor conditions, LW divergence during the nutritional challenge and convergence during recovery highlighted the sensitivity of WoW to short-term changes in nutrient supply. Under grazing conditions, restricted pasture access resulted in sustained reductions in LW, accompanied by lower milk yield and fat- and protein-corrected milk during mid-lactation.

Across experiments, LW was associated with estimated dry matter, energy, and protein intake variables; however, these relationships should be interpreted with caution, as intake was not directly measured at the individual level and was partly derived from predictive approaches. The present findings support the biological sensitivity of the WoW system to detect nutritionally induced LW variation under the controlled experimental conditions tested.

Overall, WoW systems represent a feasible approach for trajectory-based assessment of nutritional status in dairy sheep. However, the absence of direct individual intake measurements and the controlled experimental conditions, including guided animal passage through the WoW platform, should be considered when interpreting LW responses. Their effective use relies on robust data filtering and appropriate contextual interpretation, including physiological stage and production indicators. When integrated with complementary data streams, WoW systems may support the early detection of nutritional imbalances and contribute to decision-making processes in precision livestock farming systems.

Further evaluation under commercial conditions with voluntary animal traffic and potentially lower data density is required before the present findings can be fully generalised to commercial dairy sheep systems.

## Figures and Tables

**Table 1:** Chemical composition (% dry matter basis) and in vitro dry matter digestibility (IVDMD, %) of the feedstuffs used during Experiment 1. Values are means ± SE.

Feed	DM	OM	CP	EE	Starch	NDF	ADF	ADL	IVDMD
Mixed feed	88.0 ± 0.3	92.4 ± 0.3	13.8 ± 0.2	1.3 ± 0.06	19.4 ± 0.4	43.0 ± 0.5	22.5 ± 0.3	2.7 ± 0.1	76.4 ± 1.2
Concentrate	89.1 ± 0.2	88.6 ± 0.2	17.0 ± 0.2	1.9 ± 0.08	21.3 ± 0.3	32.9 ± 0.2	16.5 ± 0.2	3.2 ± 0.2	78.2 ± 0.1
Alfalfa hay	88.0 ± 0.3	91.6 ± 0.1	16.6 ± 1.2	1.1 ± 0.07	n.d.	52.2 ± 1.2	34.6 ± 0.7	7.0 ± 0.3	58.8 ± 1.0

DM: dry matter; OM: organic matter; CP: crude protein; EE: ether extract; NDF: neutral detergent fibre; ADF: acid detergent fibre; ADL: acid detergent lignin; IVDMD: in vitro dry matter digestibility; n.d.: not determined.

**Table 2:** Effects of feeding level (FL, H vs. L), experimental week and their interaction on feed intake variables in dry ewes during Experiment 1. Values are least-square means ± SE.

Week		1	2	3	4	5	Effects (*p*-Value)		
Period		Adaptation	Adaptation	Challenge	Challenge	Recovery	FL	Week	FL × Week
DMI kg/head/day	H	1.32 c	1.31 Bc	1.56 Aa	1.42 Ab	1.29 Bd	<0.001	<0.001	<0.001
	L	1.32 b	1.35 Aa	0.89 Bd	1.06 Bc	1.32 Aa			
	SE±	0.003	0.003	0.003	0.003	0.003			
UFLI UFL/head/day	H	0.98 c	0.95 Be	1.18 Aa	1.08 Ab	0.96 Bd	<0.001	<0.001	<0.001
	L	0.98 b	1.00 Aa	0.69 Bd	0.81 Bc	0.98 Ab			
	SE±	0.002	0.002	0.002	0.002	0.002			
CPI g/head/day	H	199 d	193 Be	278 Aa	254 Ab	215 Ba	<0.001	<0.001	<0.001
	L	200 c	204 Ab	158 Be	188 Bd	220 Aa			
	SE±	0.2	0.2	0.2	0.2	0.2			
MPI g/head/day	H	180 d	174 Be	249 Aa	227 Ab	193 Bc	<0.001	<0.001	<0.001
	L	180 c	184 Ab	142 Be	168 Bd	197 Aa			
	SE±	0.25	0.25	0.25	0.25	0.25			

H: high feeding level; L: low feeding level; DMI: dry matter intake; UFLI: net energy intake; CPI: crude protein intake; MPI: metabolizable protein intake. Different uppercase letters indicate significant differences between feeding levels within the same week, whereas different lowercase letters indicate significant differences among weeks within the same feeding level (*p* < 0.05).

**Table 3:** Effect of pasture access times (AT), experimental week and their interaction on herbage dry matter availability and floristic composition (proportion on DM basis) before grazing in Experiment 2. Values are least-square means ± SE.

				Effects (*p*-Value)		
	AT6	AT2	SE±	AT	Week	AT × Week
Herbage biomass (DM t/ha)	4.69	4.22	0.19	>0.05	<0.001	<0.001
Daily herbage availability (DM kg/head/day)	2.09	1.88	0.08	>0.05	<0.001	<0.001
Hourly herbage availability (DM kg/head/h)	0.35 A	0.94 B	0.03	<0.001	<0.001	<0.001
Italian ryegrass (%)	45.7 B	65.3 A	3.78	<0.01	<0.01	>0.05
Berseem clover (%)	16.2	11.9	1.92	>0.05	>0.05	<0.01
Other species (%)	38.1 A	22.9 B	3.20	<0.01	<0.001	>0.05

AT6: pasture access time of 6 h/day; AT2: pasture access time of 2 h/day. DM: dry matter; Different uppercase letters indicate significant differences between pasture access treatments within the same row (*p* < 0.05).

**Table 4:** Chemical composition (% dry matter basis) and in vitro dry matter digestibility (IVDMD, %) of the feedstuffs used in Experiment 2. Values are means ± SE.

Feed	DM	OM	CP	EE	NDF	ADF	ADL	IVDMD
Herbage AT6	27.2 ± 2.5	91.3 ± 0.3	13.2 ± 1.2	3.4 ± 0.2	48.2 ± 3.8	26.4 ± 2.8	1.4 ± 0.7	69.9 ± 7.5
Herbage AT2	27.2 ± 2.5	91.7 ± 0.3	15.0 ± 1.2	3.3 ± 0.2	46.2 ± 3.8	26.3 ± 2.8	2.6 ± 0.7	69.0 ± 7.5
Concentrate	89.0 ± 0.2	88.8 ± 0.1	17.6 ± 0.3	2.7 ± 0.01	41.8 ± 0.9	17.0 ± 0.4	3.0 ± 0.1	78.2 ± 1.0
Ryegrass hay	88.1 ± 0.7	99.0 ± 0.2	6.5 ± 0.3	2.6 ± 0.04	63.9 ± 1.5	37.0 ± 1.2	2.3 ± 0.7	54.2 ± 2.2

AT6: pasture access time of 6 h/day; AT2: pasture access time of 2 h/day. DM: dry matter; OM: organic matter; CP: crude protein; EE: ether extract; NDF: neutral detergent fibre; ADF: acid detergent fibre; ADL: acid detergent lignin; IVDMD: in vitro dry matter digestibility.

**Table 5:** Effects of pasture access time (AT), experimental week and their interaction on feed intake in lactating ewes during Experiment 2. Values are least-square means ± SE.

		Week									Effects (*p*-Value)		
Item		1	2	3	4	5	6	7	8	9	AT	Week	AT × Week
DMI, kg/head/day	AT6	2.40 Aa	2.40 Aa	2.16 Ac	2.24 Ab	2.17 Ac	1.96 Ad	1.85 Ae	1.85 Ae	1.86 Ae	<0.001	<0.001	<0.001
	AT2	2.07 Ba	1.99 Bb	1.80 Bc	1.80 Bc	1.78 Bc	1.66 Bd	1.58 Be	1.56 Be	1.56 Be			
	SE±	0.01	0.01	0.01	0.01	0.01	0.01	0.01	0.01	0.01			
UFLI, UFL/head/day	AT6	2.19 Aa	2.14 Ab	1.91 Ac	1.47 Ad	1.47 Ad	1.36 Ae	1.24 Af	1.07 Ag	1.08 Ag	<0.001	<0.001	<0.001
	AT2	1.69 Ba	1.61 Bb	1.42 Bc	1.12 Bd	1.12 Bd	1.07 Be	1.00 Bf	0.95 Bg	0.94 Bg			
	SE±	0.01	0.01	0.01	0.01	0.01	0.01	0.01	0.01	0.01			
CPI, g/head/day	AT6	335 Aa	328 Ab	311 Ac	284 Ad	285 Ad	263 Ae	240 Af	208 Ag	209 Ag	<0.001	<0.001	<0.001
	AT2	303 Ba	288 Bb	261 Bc	220 Bd	220 Bd	210 Be	196 Bf	184 Bg	183 Bg			
	SE±	2.99	2.99	2.99	2.99	2.99	2.99	2.99	2.99	2.99			
MPI, g/head/day	AT6	305 Aa	299 Ab	283 Ac	257 Ad	258 Ad	238 Ae	217 Af	189 Ag	190 Ag	<0.001	<0.001	<0.001
	AT2	274 Ba	260 Bb	236 Bc	199 Bd	199 Bd	191 Be	178 Bf	167 Bg	166 Bg			
	SE±	2.71	2.71	2.71	2.71	2.71	2.71	2.71	2.71	2.71			

AT6: pasture access time of 6 h/day; AT2: pasture access time of 2 h/day. DMI: dry matter intake; UFLI: net energy intake; CPI: crude protein intake; MPI: metabolizable protein intake. Different uppercase letters indicate significant differences between pasture access treatments within the same week, whereas different lowercase letters indicate significant differences among weeks within the same treatment (*p* < 0.05).

**Table 6:** Effects of pasture access times (AT), experimental week and their interaction on milk yield and milk composition in lactating ewes during Experiment 2. Values are least-square means ± SE.

Item		Week					Effects (*p*-Value)		
		1	3	5	7	9	AT	Week	AT × Week
Milk, kg/head/day	AT6	1.93 ab	2.00 Aa	1.83 Ab	1.32 c	1.07 d	<0.01	<0.001	<0.01
	AT2	1.81 a	1.64 Bb	1.56 Bb	1.21 c	1.09 d			
	SE±	0.05	0.05	0.05	0.05	0.05			
FPCM, kg/head/day	AT6	1.58 a	1.64 Aa	1.45 Ab	1.09 c	0.88 d	<0.05	<0.001	<0.001
	AT2	1.49 a	1.37 Bb	1.28 Bb	1.04 c	0.95 c			
	SE±	0.05	0.05	0.05	0.05	0.05			
Fat, g/kg	AT6	5.07	4.93	4.63	5.10	5.05	0.11	<0.001	0.15
	AT2	5.07	5.09	4.96	5.26	5.40			
	SE±	0.11	0.11	0.11	0.11	0.11			
Protein, g/kg	AT6	4.17	4.49	4.44	4.45	4.52	0.88	<0.001	0.36
	AT2	4.12	4.44	4.37	4.48	4.61			
	SE±	0.06	0.06	0.06	0.06	0.06			

AT6: pasture access time of 6 h/day; AT2: pasture access time of 2 h/day. FPCM: fat- and protein-corrected milk. Different uppercase letters indicate significant differences between pasture access treatments within the same week, whereas different lowercase letters indicate significant differences among weeks within the same treatment (*p* < 0.05).

**Table 7:** Linear mixed-model estimates describing the associations between live weight (LW) and intake-related variables across Experiments 1 and 2. Fixed effects included LW, initial LW, experiment, and week within experiment. Variance components and model fit statistics are reported.

Trait	Intercept (a)	SE (a)	Slope LW (b)	SE (b)	*p*-Value LW Slope	Variance (Intercept)	Variance (Residual)	Variance (Fixed Part)	Total Variance	Marginal R^2^	Conditional R^2^
DMI	1.49	0.16	0.023	0.004	<0.001	0.022	0.009	0.11	0.14	0.77	0.94
UFLI	0.75	0.15	0.024	0.003	<0.001	0.010	0.020	0.12	0.15	0.81	0.87
CPI	151.7	22.25	4.520	0.647	<0.001	383.4	290.7	1858	2532	0.73	0.88
MPI	137.9	20.28	4.073	0.582	<0.001	320.9	233.3	1546	2100	0.74	0.89

DMI: dry matter intake (kg/head/day); UFLI: net energy intake (UFL/head/day); CPI: crude protein intake (g/head/day); MPI: metabolizable protein intake (g/head/day).

**Table 8:** Linear mixed-model estimates describing associations between intake traits, live weight (LW), Milk_PC2, and week of lactation in Experiment 2. Variance components and model fit statistics are reported.

Trait	Intercept (a)	SE (a)	Slope LW (b)	SE (b)	*p*-Value LW Slope	Variance (Intercept)	Variance (Residual)	Variance (Fixed Part)	Total Variance	Marginal R^2^	Conditional R^2^
DMI	–1.57	0.56	0.020	0.013	0.146	0.316	0.050	0.530	0.896	0.59	0.94
UFLI	0.79	0.17	0.006	0.004	0.166	0.000	0.047	0.120	0.167	0.72	0.72
CPI	100.4	26.1	2.282	0.593	0.016	481.2	188.6	2148	2818	0.76	0.93
MPI	91.42	23.7	2.065	0.538	0.010	414.4	150.3	1763	2328	0.76	0.93

DMI: dry matter intake (kg/head/day); UFLI: net energy intake (UFL/head/day); CPI: crude protein intake (g/head/day); MPI: metabolizable protein intake (g/head/day).

## Data Availability

The data presented in this study are not publicly available because they are part of an ongoing study. Data are available from the corresponding author upon reasonable request.

## Supplementary Materials

The following supporting information can be downloaded at: https://www.mdpi.com/article/10.3390/s26123732/s1, Figure S1: Weekly live weight (LW) trajectories of ewes under high (H) and low (L) feeding levels during Experiment 1. Values are mean ± SEM. The figure illustrates temporal LW responses during the adaptation, challenge, and recovery phases; Figure S2: Weekly live weight (LW) trajectories of lactating ewes under two pasture access treatments (AT6: 6 h/day; AT2: 2 h/day) during Experiment 2. Values are mean ± SEM. The figure illustrates the temporal pattern of LW responses throughout the grazing period.

## Abbreviations

The following abbreviations are used in this manuscript:

E1	Experiment 1
E2	Experiment 2
WoW	Walk-over-weighing
EID	Electronic identification
LW	Live weight
FL	Feeding level
L	Low feeding level
H	High feeding level
AT6	Access time at pasture (6 h/day)
AT2	Access time at pasture (2 h/day)
W	Week
BCS	Body condition score
DM	Dry matter
OM	Organic matter
CP	Crude protein
EE	Ether extract
NDF	Neutral detergent fibre
ADF	Acid detergent fibre
ADL	Acid detergent lignin
IVDMD	In vitro dry matter digestibility
UFL	Feed units for milk production
MP	Metabolizable protein
FPCM	Fat- and protein-corrected milk
DMI	Dry matter intake
UFLI	UFL intake
CPI	Crude protein intake
MPI	Metabolizable protein intake
