# Supplementary material for: Detection of Nutritionally Driven Live Weight Changes in Dairy Ewes Using a Walk-over-Weighing System
Source: Sensors (Basel). 2026 Jun 11;26(12):3732. doi: 10.3390/s26123732 (PMC13306742; doi:10.3390/s26123732)
Supplement: Supplementary file 1 [file sensors-26-03732-s001.zip › sensors-4305273-supplementary.pdf]

Supplementary Materials

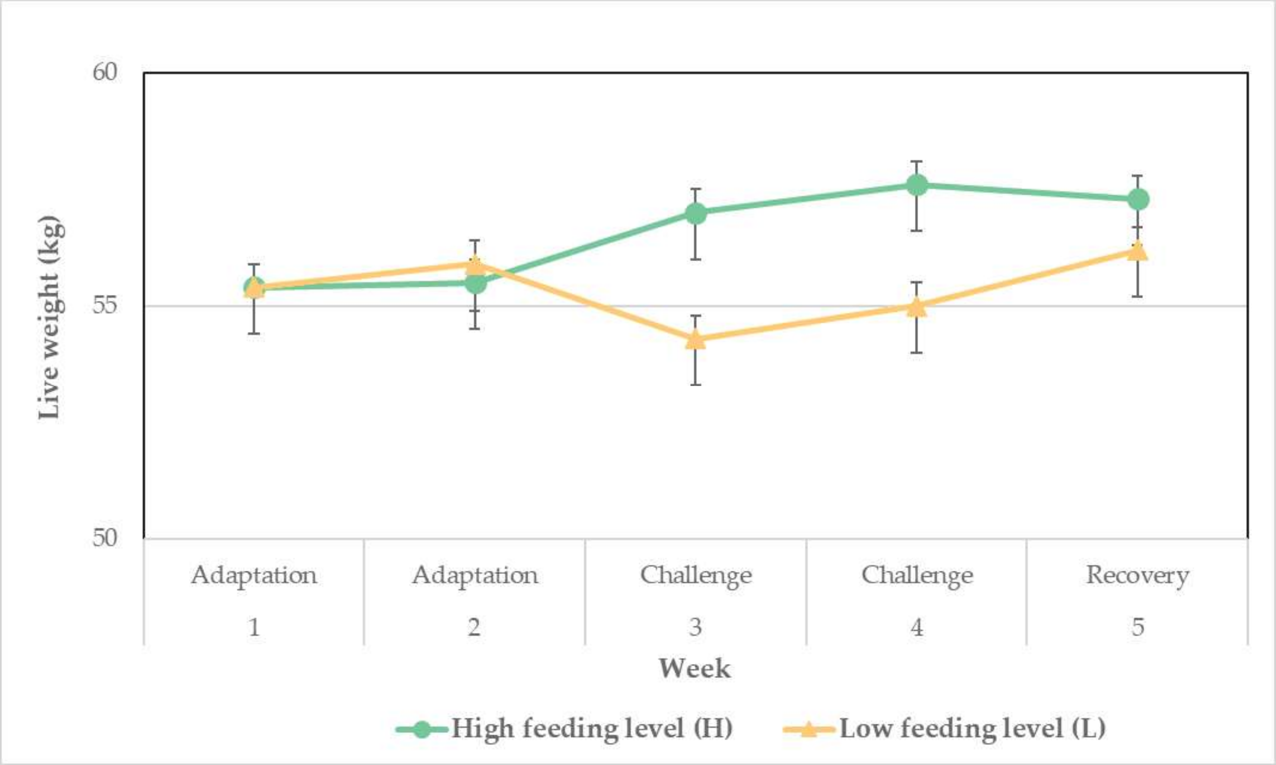

Figure S1. Weekly live weight (LW) trajectories of ewes under high (H) and low (L) feeding levels during Experiment 1. Values are mean ± SEM. The figure illustrates temporal LW responses during the adaptation, challenge, and recovery phases.

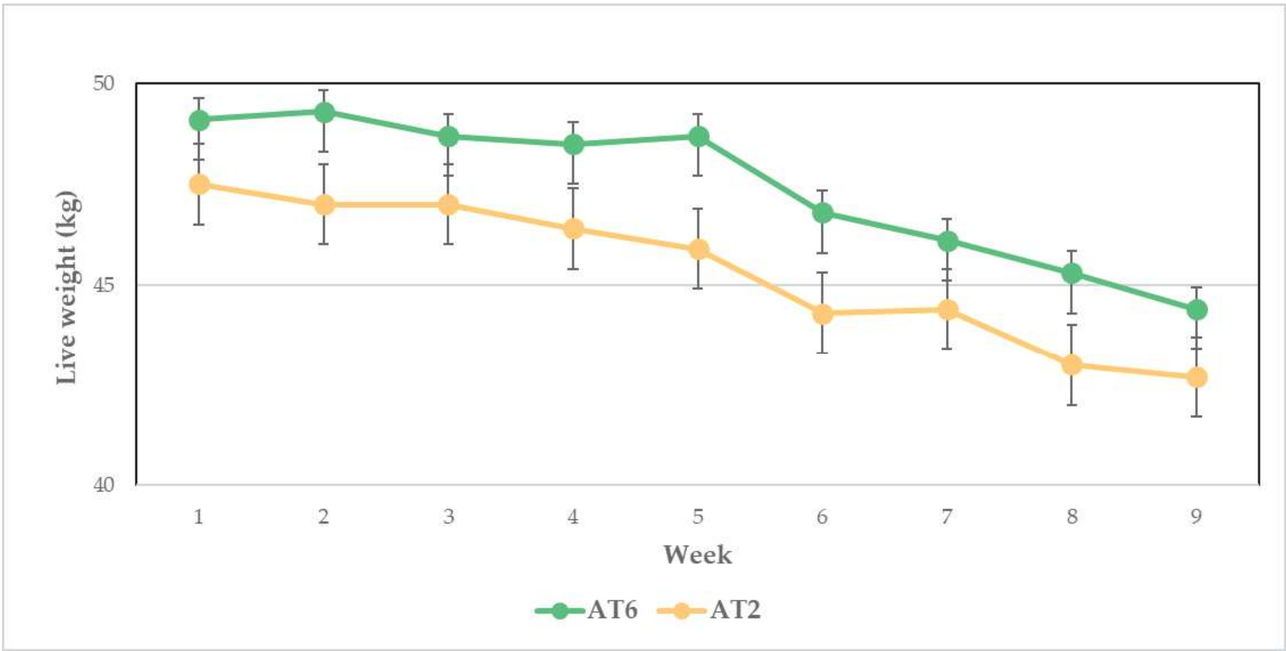

Figure S2. Weekly live weight (LW) trajectories of lactating ewes under two pasture access treatments (AT6: 6 h/day; AT2: 2 h/day) during Experiment 2. Values are mean ± SEM. The figure illustrates the temporal pattern of LW responses throughout the grazing period.
